# Gleanings from Our Exchanges

**Published:** 1873-07-15

**Authors:** 


### Gleanings from Our Exchanges.

THE CAUSES AND NATURE OF HYDROCELE. — Every clinician has met with cases of lesion of the testicle accompanied with hydrocele, but they have all considered the lesions not as a cause of the hydrocele, but as a complication or a consequence of the compression exercised by the liquid about the testicle. In the author's opinion, based upon eight observations of cases occuring *au hazard* in the same year, the testicle is far from being absolutely sound in simple hydrocele, for in all the cases epididymus was more or less indurated. In some, the body of the testicle showed a certain augmentation in volume and consistence, and was at the same time painful to pressure:

It should be added that no one of the patients had had orchitis; they were all free from blemish by syphilis, blenorrhagia or phthisis; in short, the testicle upon the opposite side was perfectly sound. There was manifest thus, a kind of partial epididymus, most frequently alone; more rarely accompanied by a swelling in the body of the testicle. This epididymus is always sub-acute; indolent, and but little painful; yet there was always enough inflammation present to cause an abundant effusion from the tunica vaginalis.

What, then, is the cause of this orchitis, which engenders the hydrocele considered spontaneous? If attention be paid to these cases, it will be seen that the majority of the individuals affected with hydrocele, have passed the fourth, even the fifth decade of life. At this age lesions of the neck of the bladder, and of the prostate are common, (prostatic calculi, uric gravel, hypertrophy of the prostate, varicose condition of the neck of the bladder, etc.) Thus, there exists in advanced life a low form of irritation about these parts, which extends to the epididymus and testicle; and is the first cause of orchitis. — *Panas, Arch. Gen. de Med.* — *France Medicale, June 7th*, 1873.

TREATMENT OF CONSUMPTION AT THE BROMPTON HOSPITAL. — T. G. Roddick, M.D., House Surgeon to Montreal General Hospital, writing from London to the *Canada Medical and Surgical Journal*, says that two thousand gallons of cod-liver oil are consumed in that institution in a year. Lead, turpentine and gallic acid are the sheet anchors there in hæmoptysis. Although the physicians are beginning to use ergotine hypodermically with moderate success, they do not depend on the latter remedy in severe cases. — *Medical Record.*

Successful Transplantation of a Rabbit's Conjunctiva, and its Adaptation to the Human Eye. — J. R. Wolfe, of Glasgow, reports the case of a foundryman, thirty-one years of age, where, in consequence of a severe burn from a mass of red-hot iron, there had resulted an extensive symblepharon, glueing the lid firmly to the ball, in such a position that its ciliary border covered the upper edge of the pupil. Six weeks after the injury, when the inflammation had entirely subsided, Dr. W. dissected the lower lid from its attachments to the ball, and, to prevent its re-adhering, sutured, on the raw surface thus produced, the conjunctiva which he then dissected from the eye of a rabbit. The animal was under the influence of chloroform, and the part selected was that covering the nictitating membrane. The eye was dressed with dry charpie and a compressive bandage. On the day following, the transplanted conjunctiva had a grayish aspect, and warm fomentations were ordered. On the second day the eye was swollen and very painful, but the conjunctiva had lost its gray hue and become vascular. On the eighth day, he was discharged, cured; the eye-ball freely movable, and the transplanted conjunctiva healthy and adherent throughout. A few days subsequently, an iridectomy was successfully performed at the upper, inner quadrant of the cornea, with the result of restoring him to useful eyesight. The patient was seen two months subsequently, and the condition of the eye was still entirely satisfactory. The author then gives a second, somewhat similar case, with equally favorable result.— *Glasgow Med. Jour.*, 1873.— *Philadelphia Times*, *June* 21*st*, 1873.

Convulsions treated by Chloroform. — In confirmation of the experience of Mr. Mowatt, recorded in the *Journal* of May 31st, I may mention that some years ago, in a severe case of convulsions in a child aged seven months, I tried the chloroform treatment, and finding it beneficial, have continued it to the present time, with the best and most successful results. I only use the warm bath, etc., where I dare not treat with chloroform. When I am called to a case of convulsions, the first thing I do is to administer chloroform, and keep the patient under its influence until the convulsions have passed away. When the child wakes, I give small doses of bromide of potassium, taking care that the bowels are freely opened. I fully agree with Mr. Mowatt as to the caution required in treating cases where disease of the brain is at work. — *Frazer*. — *British Med. Journal*, *June* 14*th*, 1873.

Treatment of Psoriasis.—We observe in a recent number of the *British Medical Journal* that that leading dermatologist, Dr. Tilbury Fox, thinks that the treatment of psoriasis by arsenic internally, and tarry preparations externally, is erroneous, and much too generally employed. For his own part, he has almost entirely given up this plan of treatment, except in certain chronic cases, and where there is a syphilitic taint, when he found Donovan's solution of great value. In all other cases, he relies mainly upon soothing applications locally, viz. : wet packing, alkaline and sitz baths, and oily preparations ; and internally, remedies in accordance with the constitutional diathesis. This plan of treatment is especially successful in acute cases occurring in young children. Dr. Fox lays great stress upon psoriasis being treated on the same principles as other cases, with due regard to constitutional and other causes likely to affect and modify it.—*Medical and Surgical Reporter.*

The Employment of Pessaries. John Lambert, M.D., Salem, N. Y., (*Journ. Gynæcological, Soc.*,) in a communication on "Uterine Displacements," says that the use of pessaries in these cases complicates treatment, and hazards successful results. In a certain proportion of cases of uterine displacements, a comparatively small number of well-fitting pessaries, in the hands of intelligent and skillful gynæcologists, are essential to the cure, not only of the mal-position, but also of the abnormal condition of the organ which accompanies it. A pessary, of whatever kind, when employed for the mechanical support of the uterus, is a foreign body, liable so do serious, and perhaps fatal mischief, and never should be placed in situ without great circumspection on the part of the physician. It has no miraculous power, and its potency for harm is very much underrated.

He is of the opinion that intra-uterine, or stem pessaries, should seldom be used. Of the various forms of intra-vaginal pessaries in use, he prefers and employs the closed lever made of hardened rubber, and the soft ring made of delicate elastic strips of fine whalebone, covered with pure rubber, made by Tieman & Co. — *Medical Record.*

A mixture of pulv. steatite (soapstone) two parts, and hyd. chlor. mitis one part, is the most elegant and effective dry application to the chafed skin of infants. Dry hyd. chlor. mitis, applied once or twice a day to tumid and hemorrhoids situated about the anus, rarely fails to cure them in a few days. —*Dr. A. S. Hudson, in the Pacific Medical and Surgical Journal.*

Chronic Eczema of the Extremities. — Dr. MacCallum, of the Montreal General Hospital (*Canada Med. and Surg. Jour.*, Nov. 1872), successfully treated an obstinate case of this affection, the patient, male, being forty-two years old, as follows: A solution of potassa caustica gr. v, to water ℥ ij, was used outwardly; being brushed lightly over the eruption once daily. The following mixture was ordered internally: ℞. hydrarg. bichlor. gr. iv; liq. arsenic, ♏xv; acid hydrochlor. dil. ʒ iss; aquæ ad ℥ viij; M. — ℥ ss, ter die. — *Medical Record.*

Excision of Superior Maxillary. — John P. Wall, M.D., of Tampa, Florida (*Atlanta Med. and Surg. Journal*), records a case of excision of the superior maxillary bone; the patient being a female child of eight years. The chief interest of the case consisted in the absence of any kind of pain, the rapidity of the growth without known cause, and the rarity of such a diseased condition of this bone in children.

Carbolic Acid in Obstinate Leucorrhœa. —Dr. Whitaker, of Cincinnati (*Cincinnati Lancet and Observer*), speaks highly of carbolic acid in the treatment of obstinate leucorrhœa. The application is made upon the cotton - wrapped sound. The instrument, so enveloped, is first introduced and the uterine surface cleansed; a new layer of cotton is substituted, medicated by immersion in a solution of proper strength, and applied to every portion of the uterine cavity.

The London Medical Societies. — The presidents of the various medical societies of London, for the year 1873, are as follows: Pathological Society, Sir Wm. Jenner; Obstetrical Society, Dr. Edward J. Tilt; Harverian Society, Dr. T. Ballard; Royal Medical and Chirurgical Society, Dr. C. J. B. Williams; Clinical Society, Prescott G. Hewett; Medical Microscopical Society, Mr. Jabez Hogg.

Ligation of Right Carotid Artery. — Dr. Pigne, Dupuytren (*Pacific Med. Jour.*), exhibited to the San Francisco Medical Socity a patient on whom he had successfully performed the ligation of the right carotid artery for aneurism. The ligature came away on the seventeenth day. In five months the tumor had entirely disappeared.

The Raising of Children Doubled. — Dr. Farr reports that the proportion of children raised has doubled within a hundred years. In London, the proportion of deaths under five years were — 1730 to 1749, 74.5; per cent.; 1770 to 1789, 51.5 per cent.; 1851 to 1870, 29.8 per cent.

